# Improving soil physical properties and cassava productivity through organic manures management in the southern Cameroon

**DOI:** 10.1016/j.heliyon.2022.e09570

**Published:** 2022-06-02

**Authors:** Eloi Gervais Bilong, Monique Abossolo-Angue, Francis Ngome Ajebesone, Bienvenu Désiré Anaba, Birang À. Madong, Lucien Bidzanga Nomo, Paul Bilong

**Affiliations:** a Institute of Agricultural Research for Development (IRAD), P.O. Box 2123, Messa-Yaounde, Cameroon; b Department of Earth Sciences, Faculty of Science, University of Yaounde 1, P. O. Box 812, Yaounde, Cameroon

**Keywords:** *Tithonia diversifolia*, Fresh biomass, Poultry manure, Soil properties, Cassava

## Abstract

Cassava cultivation causes serious soil fertility depletion in Cameroon. Organic manures can contribute to maintaining the productivity and sustainability of cassava production systems due to their availability and cheapness. This study was conducted during two successive cropping seasons (2016/2017 and 2017/2018) in the Southern Cameroon, to determine the effects of organic manures on soil physical properties, and cassava growth and yield. *Tithonia diversifolia* fresh biomass (TB) and poultry manure (PM) were used as organic manures. Different treatments were used, namely: (i) control (no amendment), (ii) mineral fertilizer (MF (450 kg ha^−1^ 13-13-23 NPK + 100 kg ha^−1^ Urea)), (iii) two rates of TB (applied at 10 and 20 t ha^−1^), (iv) two rates of PM (applied at 10 and 20 t ha^−1^) and (v) two rates of the mixture of TB and PM (applied at 5 and 10 t ha^−1^ each). The experimental designed was randomized complete block with three replicates per treatment. The application of TB, PM and TB + PM (mixture) lowered bulk density by 14–22.6%, 21.5–26.2% and 18.2–25%, respectively. While total porosity and water holding capacity were increased with manure application by 9.8–15.1% and 13.3–30.3%, respectively. The mineral fertilizer had no significant effect on these soil physical parameters. Application of TB and PM either solely or mixed significantly improved growth of cassava. Stem height, stem diameter and canopy diameter increased by 34.3–60%, 17.4–30.4% and 19.7–32.7%, respectively. Organic manures performed better than the mineral fertilizer. Aboveground dry biomass and fresh tubers yield increased by 21.7–59.6 and 76.6–112.2% with organic manures, and by 23.2 and 68.5% with mineral fertilizer, respectively. The best performance of cassava in terms of fresh tubers yield (51.78 t ha^−1^) occurred with the mixture of TB and PM applied at 10 t ha^−1^ each. The study revealed that fresh biomass of *Tithonia diversifolia* and poultry manure can be used to improve cassava production and sustain soil productivity in the humid forest zone of Southern Cameroon.

## 1 Introduction

Being one of the most important crops in tropical areas (Scott et al., 2000), Cassava (*Manihot esculenta crantz*) is a major staple food crop in most parts of Africa; thus playing an important role in terms of food security, employment and source of income for many families (Spencer and Ezedinma, 2017). It is also important for the fact that it produces a lot of starchy tuberous root, which is a valuable source of cheap calories especially in developing countries (Dixon et al., 2002). Cassava is an important industrial raw material for the production of starch, alcohol, pharmaceuticals, gums, confectioneries and livestock feed (Nnodu et al., 2006). The leaves and tender shoots are also consumed as vegetables in most part of Africa (Eke-Okoro and Dixon, 2000).

In Cameroon, Cassava is one of the most important food and cash crops; It is grown in all the five agro-ecological zones of the country (Temegne and Ngome, 2017). In the year 2014, the production of cassava roots was 4 917 544 tons (FAO, 2017). About 80% of the production came from the humid forest zone of the country (MINADER, 2012). Cameroon economy remains highly dependent on its agricultural sector, and employs more than 68% of the national active labour force, which contributes significantly to the GDP (Gross Domestic Product) and contributes in about 15% of public budget (Fonji et al., 2017).

Cassava is cultivated by 75% smallholder farmers in the humid forest zone of Cameroon (Billa et al., 2018). However, yields are very low and generally less than 17 ton·ha^−1^ compared to potential yield of 30–40 ton·ha^−1^ of improved cassava varieties available in the country (MINADER, 2012). Soils of the humid forest zone of Southern Cameroon are acidic (pH < 5) and characterized by low soil organic matter and base saturation which results in low water and nutrient retention capacity (Ngome et al., 2013). Continuous cassava cultivation without adequate soil nutrient management, mostly in the humid forest zone of Southern Cameroon, has led to continues decline of soil productivity; even though cassava can also grow on low fertile soils (Bilong et al., 2017; Ngome et al., 2013).

Cassava cultivation can cause serious soil fertility depletion due to the excess nutrient removal by the crop (Howeler, 2017). Therefore, the use of chemical fertilizers can be suitable for improving crop yield; but because of their high cost and scarcity the use of chemical fertilizers is very limited by smallholder farmers, especially in the Southern part of Cameroon (Temegne and Ngome, 2017; Kintche et al., 2017). Associated problems related to the use of chemical fertilizers are soil acidification, pollution and the lack of organic matter to improve soil physical properties (Adekiya, 2019).

Organic manures have been recommended as alternative to mineral fertilizers for improving soil productivity in agricultural systems in Africa because of their beneficial effects on soil productivity (Harraq et al., 2021; Ravindran et al., 2017; Adekiya and Agbede, 2016). Their effects include improvement of soil physical and chemical properties and crops yield (Al-Gaadi et al., 2019; Biratu et al., 2019).

Very few studies on cassava fertilization have been conducted in Cameroon. Temegne and Ngome (2017) evaluated the effect of mineral fertilizers and poultry manure on cassava growth and yield in the Centre Region of Cameroon. Likewise, Bilong et al. (2017) evaluated the effect of *Tithonia diversifolia* green biomass on the performance of cassava in the forest zone of Cameroon. With these studies, there is yet limited understanding of the impact of fertilization on soils under cassava cultivation. The aim of this study was to evaluate the effects of *Tithonia diversifolia* fresh biomass and poultry manure application on soil physical properties, and cassava growth and yield by testing the hypothesis that, the use of organic manures may improve cassava production and sustain soil productivity in the humid forest zone of Southern Cameroon.

## 2 Materials and methods

### 2.1 Study sites

The study was carried out during two successive cropping seasons (2016/2017 and 2017/2018) in a locality named Essong-mintsang (04°05′02″N Latitude and 11°35′09″E Longitude) in Obala, Lekie division in the humid forest zone of southern Cameroon. The area receives between 1200 and 1500 mm of rain annually in a bimodal distribution (Hauser et al., 2008), such that the major and minor rainy seasons last from mid-March to early July and from mid-August to mid-November, respectively (Tchatat et al., 2004). The mean temperature is between 23 and 26 °C. The locality Essong-Mintsang lies in the forest savannah transitional zone of the rainforest region and now has a more or less semi-deciduous type of vegetation instead of primary evergreen forest. The soil profile described in the site belongs to ferralsols group according to WRB (2014), with sandy clay texture. The moderate bulk density (1.2 g cm^−3^), total porosity (54.7%) and water holding capacity (37.5%) of this soil reveal its suitability to roots penetration and cassava cultivation. Chemical analysis of *Tithonia diversifolia* fresh biomass and poultry manure have shown that poultry manure has higher pH compared to *Tithonia diversifolia* fresh biomass. Among the two organic fertilizers, *Tithonia diversifolia* fresh biomass has higher content of N, K and Ca. Whereas, poultry manure has higher C and P concentrations and C/N ratio (Table 1).

**Table 1:** Chemical properties of the organic manures used.

Organic manures		
Chemical parameters	Tithonia fresh biomass	Poultry manure
Carbon (%)	24.8	36.2
Nitrogen (%)	3.47	2.53
C/N ratio	7.15	14.3
Phosphorus (%)	0.6	1.3
Potassium (%)	3.8	2.1
Calcium (%)	3.06	1.52
Magnesium (%)	0.54	0.6

### 2.2 Experimental design

The experiment was laid out in randomized complete block design (RCBD) replicated in three blocks with 8 treatments. Each block was divided in eight plots of 16m^2^ giving a total of 48 plots covering a surface area of 768m^2^. *Tithonia diversifolia* fresh biomass (TB) and poultry manure (PM) were used as organic fertilizers. They were chosen as soil amendment because of their high nutrient contents (Adekiya, 2019; Bilong et al., 2017; Hafifah et al., 2016). The amendments consisted of two rates of *Tithonia divesrifolia* fresh biomass (TB) and poultry manure (PM) either solely or mixed, a control without amendment and a dose of mineral fertilizer (MF) to give a total of 08 treatments; which are: Control (no amendment), MF (450 kg ha^−1^ 13-13-23 NPK + 100 kg ha^−1^ Urea), TB applied at 20 t ha^−1^, PM applied at the rate of 20 t ha^−1^, TB + PM applied at 10 t ha^−1^ each, TB applied at 10 t ha^−1^, PM applied at 10 t ha^−1^, and TB + PM applied at 5 t ha^−1^ each. MF (450 kg ha^−1^ 13-13-23 NPK + 100 kg ha^−1^ Urea) was used as equivalent of 100N:22P:83 K kg·ha^-1^ which is the mineral fertilizer rate recommended by Howeler et al. (2017) for cassava production. Urea was applied in two split doses: 50 kg ha^−1^ at planting and 50 kg ha^−1^ three months after planting (MAP). NPK (13-13-23) fertilizer was divided in two splits doses: 225 kg ha^−1^ applied one month after planting (MAP) and 225 kg ha^−1^ three MAP.

Poultry manure (faeces of chickens with mixture of bedding material) and *Tithonia diversifolia* fresh biomass (fresh leaf with petiole and soft stem) were collected locally. The plant material used in this study was the improved cassava variety 8034 collected from the Institute of Agricultural Research for Development (IRAD). Land preparation was done manually by ploughing using a hoe. The organic manures (TB and PM) were incorporated into the soil at 20 cm depth two weeks before planting. Cassava cuttings of 25 cm in length were planted at 1 × 1m inter and intra spacing. Manual weeding was done as required, until the plant canopy was established.

Before incorporation of organic fertilizers into the plots, samples were collected from each manure for chemical analysis (C, N, P, K, Ca and Mg) as described by Tel and Hagarty (1984). Total N was determined by the Kjeldahl digestion method. Ground samples were digested with nitric-perchloric-sulphuric acid mixture for determination of P, K, Ca and Mg. Phosphorus was determined by colorimetry using the vanadomolybdate method, K was determined by a flame photometer and Ca and Mg were determined by using the EDTA titration method. The organic carbon (OC) content was determined by the Walkley and Black procedure using the dichromate wet oxidation method.

Soil samples were collected from the topsoil at 20 cm depth by the use of an auger before the establishment of the study and 12 months after planting. Bulk density (BD), particle size distribution and water holding capacity (WHC) were analysed from these soil samples. The soil particle size analysis was determined by pipette method (Gee and Bauders, 1986), the bulk density was determined by the core method (Blake and Hartge, 1986) and the water holding capacity (WHC) was determined following the method of ISO 11274 (2019).

The growth and yield data of cassava were obtained from randomly selected six plants per plot. The stem height (SH), stem diameter (SD) and canopy diameter (CD) were measured as growth parameters; and the aboveground dry biomass (ADB) and fresh tuber yield (FTY) measured as yield parameters. All the measurements were performed 12 MAP.

### 2.3 Statistical analyses

Analysis of variance (ANOVA) was performed from the data collected, and the means of treatment were compared using the Duncan's multiple range test at P ≤ 0.05. Pearson's correlation was used to evaluate the correlation between soil physical properties and cassava growth and yield. All the statistical analysis were performed by the use of the Statistical Package for the Social Sciences (SPSS) version 17.0 (Chicago: SPSS Inc., 2008).

## 3 Results

### 3.1 *Tithonia diversifolia* fresh biomass and poultry manure effects on soil physical properties

The soil physical properties (bulk density, total porosity and water holding capacity) for the different treatments applied are shown in Table 2. Application of *Tithonia diversifolia* fresh biomass (TB) and poultry manure (PM), solely or mixed decreased soil bulk density, improved total porosity and water holding capacity compared to the unamended control. Soil water holding capacity and total porosity increased as organic manure rates increased, while soil bulk density decreased with increased manure rates. The lowest bulk density, the highest total porosity and the highest water holding capacity occurred with PM applied at 20 t ha^−1^ and TB + PM applied at 10 t ha^−1^ each. Average over the cropping seasons (Table 3), applications of TB and PM, solely or mixed at different rates reduced soil bulk density by 14–26%, increased total porosity by 10–16% and water holding capacity by 13–30%, compared to the control. Application of mineral fertilizer (MF) had no significant effect on soil physical parameters. Significant changes were noticed on soil physical properties for both cropping seasons (2016/2017 and 2017/2018) but were better improved in the second cropping season (2017/2018) compared to the first (2016/2017). Taken as individual factors, cropping season (S) was not significant for bulk density (BD), total porosity (TP) and water holding capacity (WHC). Treatment (T) was significant for all studied soil physical parameters (BD, TP and WHC). The S × T interaction was not significant for soil bulk density, total porosity and water holding capacity.

**Table 2:** Effect of treatments on soil physical properties in 2016/2017 and 2017/2018 cropping seasons.

Cropping seasons	Treatments	BD (g·cm^−3^)	TP (%)	WHC (%)
2016/2017	NA (Control)	1.25^a^	52.8^c^	36.1^f^
	MF	1.3^a^	50.8^c^	34.8^f^
	20 t ha^−1^ TB	1.1b^c^	58.6^ab^	41.9^bc^
	20 t ha^−1^ PM	1.05^c^	60.6^a^	45.3^a^
	TB + PM 10 t ha^−1^ each	1.08^c^	59.1^ab^	43.4^b^
	10 t ha^−1^ TB	1.16^b^	56.4^b^	37.8^e^
	10 t ha^−1^ PM	1.08^c^	59.2^ab^	40^d^
	TB + PM 5 t ha^−1^ each	1.13^bc^	57.5^b^	40.9^cd^
2017/2018	NA (Control)	1.31^a^	50.4^d^	31.8^g^
	MF	1.33^a^	49.9^d^	31.9^g^
	20 t ha^−1^ TB	1.03^bc^	61.1^ab^	46.1^c^
	20 t ha^−1^ PM	1.0^d^	62.1^a^	52.3^a^
	TB + PM 10 t ha^−1^ each	0.99^d^	62.5^a^	49.4^b^
	10 t ha^−1^ TB	1.11^b^	58^c^	40.5^f^
	10 t ha^−1^ PM	1.06^c^	60.1^b^	43.6^d^
	TB + PM 5 t ha^−1^ each	1.07^cd^	59.5^bc^	42.4^e^
Treatment		∗	∗	∗
Cropping season		ns	ns	ns
Cropping season x Treatment		ns	ns	ns

Values followed by similar letters under the same column are not significantly different at p = 0.05 according to Duncan's multiple range test. BD = bulk density, TP = total porosity, WHC = water holding capacity, NA = no amendment (control), TB = *Tithonia diversifolia* fresh biomass, PM = poultry manure, MF = mineral fertilizer (450 kg ha^−1^ 13-13-23 NPK + 100 kg ha^−1^ Urea). Note: ∗, Significant difference at p = 0.05; ns, not significant at p = 0.05.

**Table 3:** Means and improvement rates of soil bulk density, total porosity and water holding capacity under different treatments over 2016/2017 and 2017/2018 cropping seasons.

Treatments	BD (g·cm^−3^)	IR (%)	TP (%)	IR (%)	WHC (%)	IR (%)
NA (Control)	1.3^a^	-	51.6^d^	-	34^e^	-
MF	1.3^a^	0	50.4^d^	-2.4	33.4^e^	-1.8
20 t ha^−1^ TB	1.06^cd^	-22.6	59.9^ab^	13.9	44a^bc^	22.7
20 t ha^−1^ PM	1.03^d^	-26.2	61.4^a^	16	48.8^a^	30.3
TB + PM 10 t ha^−1^ each	1.04^d^	-25	60.8^a^	15.1	46.4^ab^	26.7
10 t ha^−1^ TB	1.14^a^	-14	57.2^c^	9.8	39.2^d^	13.3
10 t ha^−1^ PM	1.07^cd^	-21.5	59.7^c^	13.6	41.8^cd^	18.7
TB + PM 5 t ha^−1^ each	1.1^ab^	-18.2	58.5^bc^	11.8	41.7^cd^	18.5

Values followed by similar letters under the same column are not significantly different at p = 0.05 according to Duncan's multiple range test. BD = bulk density, TP = total porosity, WHC = water holding capacity, NA = no amendment (control), TB = *Tithonia diversifolia* fresh biomass, PM = poultry manure, MF = Mineral fertilizer (450 kg ha^−1^ 13-13-23 NPK + 100 kg ha^−1^ Urea), IR = improvement rate.

### 3.2 *Titthonia diversifolia* fresh biomass and poultry manure effects on growth parameters of cassava

It was observed that application of organic fertilizer affected cassava growth parameters (Table 4). Application of *Tithonia diversifolia* fresh biomass and poultry manure, solely or combined increased stem height (SH), stem diameter (SD) and canopy diameter (CD) in the two cropping seasons (2016/2017 and 2017/2018) compared to the control and mineral fertilizer (MF) treatments. TB + PM applied at 10 t ha^−1^ each recorded the highest growth. Averaged over the two cropping seasons, stem height, stem diameter and canopy diameter of cassava increased by 34–60%, 17–30% and 20–33%, respectively, with the application of organic fertilizers compared to the control treatment (Table 5). Likewise, application of mineral fertilizer increased SH, SD and CD by 22, 9 and 21%, respectively, compared to the control. Significant changes were noticed on cassava stem height, stem diameter and canopy diameter from both cropping seasons (2016/2017 and 2017/2018) and cassava stem height increased in the first cropping season (2016/2017), while stem diameter and canopy diameter increased in the second cropping season (2017/2018). Taken as individual factors, cropping season (S) was not significant for cassava stem height (SH), stem diameter (SD) and canopy diameter (CD). Treatment (T) was significant for all these growth parameters (SH, SD and CD). The S × T interaction was not significant for cassava stem height (SH), stem diameter (SD) and canopy diameter (CD).

**Table 4:** Effect of *Tithonia diversifolia* green manure and poultry manure on cassava growth parameters in the 2016/2017 and 2017/2018 cropping seasons.

Cropping seasons	Treatments	SH (cm)	SD (cm)	CD (m)
2016/2017	NA (Control)	191.7^e^	2.3^c^	1.49^e^
	MF	220^d^	2.4^c^	1.75^cd^
	20 t ha^−1^ TB	266.7^b^	3^ab^	1.82^bc^
	20 t ha^−1^ PM	275^bc^	2.8^ab^	1.9^ab^
	TB + PM 10 t ha^−1^ each	291.7^a^	3.1^a^	1.95^a^
	10 t ha^−1^ TB	245^c^	2.9^ab^	1.3^bc^
	10 t ha^−1^ PM	237^c^	2.8^ab^	1.7^d^
	TB + PM 5 t ha^−1^ each	247^c^	2.9^ab^	1.81^bc^
2017/2018	NA (Control)	176.7f	2.2^e^	1.46^d^
	MF	233.3^e^	2.5^d^	1.8^bc^
	20 t ha^−1^ TB	278^c^	2.8^ab^	1.93^a^
	20 t ha^−1^ PM	290^b^	2.8^ab^	1.85^b^
	TB + PM 10 t ha^−1^ each	306.7^a^	3^a^	1.95^a^
	10 t ha^−1^ TB	269^cd^	2.7^bcd^	1.74^c^
	10 t ha^−1^ PM	263.3^d^	2.6^cd^	1.83^b^
	TB + PM 5 t ha^−1^ each	261.7^d^	2.8^bc^	1.82^b^
Treatment		∗	∗	∗
Cropping season		ns	ns	ns
Cropping season x Treatment		ns	ns	ns

Values followed by similar letters under the same column are not significantly different at p = 0.05 according to Duncan's multiple range test. NA = no amendment (control), TB = *Tithonia diversifolia* fresh biomass, PM = poultry manure, MF = mineral fertilizer (450 kg ha^−1^ 13-13-23 NPK + 100 kg ha^−1^ Urea), SH = stem height, SD = stem diameter, CD = canopy diameter. Note: ∗, Significant difference at p = 0.05; ns, not significant at p = 0.05.

**Table 5:** Means and improvement rates of stem height, stem diameter and canopy diameter of cassava under different treatments over 2016/2017 and 2017/2018 cropping seasons.

Treatments	SH (cm)	IR (%)	SD (cm)	IR (%)	CD (m)	IR (%)
NA (Control)	184.2^e^	-	2.3^e^	-	1.47^d^	-
MF	226.7^d^	22.2	2.5^d^	8.7	1.78^c^	21.1
20 t ha^−1^ TB	272.1^b^	45.8	2.9^ab^	26.1	1.87^b^	27.2
20 t ha^−1^ PM	282.5^b^	51.3	2.8^bc^	21.7	1.88^b^	27.9
TB + PM 10 t ha^−1^ each	299.2^a^	60	3.0^a^	30.4	1.95^a^	32.7
10 t ha^−1^ TB	257.0^c^	38	2.8^bc^	21.7	1.79^c^	21.8
10 t ha^−1^ PM	250.0^c^	34.3	2.7^c^	17.4	1.76^c^	19.7
TB + PM 5 t ha^−1^ each	254.2^c^	36.5	2.8^bc^	21.7	1.82^bc^	23.8

Values followed by similar letters under the same column are not significantly different at p = 0.05 according to Duncan's multiple range test. NA = no amendment (control), TB = *Tithonia diversifolia* fresh biomass, PM = poultry manure, MF = Mineral fertilizer (450 kg ha^−1^ 13-13-23 NPK + 100 kg ha^−1^ Urea), SH = stem height, SD = stem diameter, CD = canopy diameter, IR = improvement rate.

### 3.3 *Titthonia diversifolia* fresh biomass and poultry manure effects on yield parameters of cassava

Yield parameters of cassava were significantly affected by the amendments applied (Table 6). Applied solely or mixed, *Tithonia diversifolia* fresh biomass and poultry manure improved ADB and FTY compared to the control. Similarly, treatment with mineral fertilizer (MF) increased ADB and FTY of cassava compared to the control. A general trend of increase in yield parameters were noticed by increasing the rate of organic fertilizers applied during both cropping seasons. The highest yield occurred with the application of TB at 20 t ha^−1^, PM at 20 t ha^−1^ and both combined at 10 t ha^−1^ each. The application of *Tithonia diversifolia* fresh biomass and poultry manure, solely or combined at equal proportion, increased ABD and FTY by 22–60% and 77–172%, respectively, compared to the control. Application of mineral fertilizer increased ABD and FTY by 23 and 69% compared to the control (Table 7). Significant changes were also noticed on above ground dry biomass and fresh tuber yield of cassava from 2016/2017 and 2017/2018 cropping seasons. The second cropping season (2017/2018) performed better in terms of cassava aerial dry biomass and fresh tuber yield. Taken as individual factors, cropping season (S) was not significant for above ground dry biomass (ADB) and fresh tuber yield (FTY). Treatment (T) was significant for all yield parameters (ADB and FTY). The S × T interaction was not significant for cassava aerial dry biomass and cassava fresh tuber yield.

**Table 6:** Effect of *Tithonia diversifolia* green manure and poultry manure on cassava yield parameters in the 2016/2017 and 2017/2018 cropping seasons.

Cropping seasons	Treatments	ADB (t·ha^−1^)	FTY (t·ha^−1^)
2016/2017	NA (Control)	20.3^d^	19.6^f^
	MF	24.6^bc^	30.1^e^
	20 t ha^−1^ TB	30.7^a^	45.1^b^
	20 t ha^−1^ PM	26.4^b^	38.1^b^
	TB + PM 10 t ha^−1^ each	31.4^a^	51.3^a^
	10 t ha^−1^ TB	24.3bc	33.4^d^
	10 t ha^−1^ PM	22.9cd	30.8^e^
	TB + PM 5 t ha^−1^ each	26.4^b^	35.6^d^
2017/2018	NA (Control)	18.6^e^	16.7^d^
	MF	23.8^d^	32.9^c^
	20 t ha^−1^ TB	30.5^a^	49.9^a^
	20 t ha^−1^ PM	27.2^b^	41.2^b^
	TB + PM 10 t ha^−1^ each	31.7^a^	52.2^a^
	10 t ha^−1^ TB	25.2^cd^	35.9^c^
	10 t ha^−1^ PM	24.9^cd^	35.4^c^
	TB + PM 5 t ha^−1^ each	26.3^bc^	39.8^b^
Treatment		∗	∗
Cropping season		ns	ns
Cropping season x Treatment		ns	ns

Values followed by similar letters under the same column are not significantly different at p = 0.05 according to Duncan's multiple range test. NA = no amendment (control), TB = *Tithonia diversifolia* fresh biomass, PM = poultry manure, MF = mineral fertilizer (450 kg ha^−1^ 13-13-23 NPK + 100 kg ha^−1^ Urea), ADB = aboveground dry biomass, FTY = fresh tubers yield. Note: ∗, Significant difference at p = 0.05; ns, not significant at p = 0.05.

**Table 7:** Means and improvement rates of aboveground dry biomass and fresh tubers yield of cassava under different treatments over 2016/2017 and 2017/2018 cropping seasons.

Treatments	ADB (t·ha^−1^)	IR (%)	FTY (t·ha^−1^)	IR (%)
NA (Control)	19.5^e^	-	18.1^f^	-
MF	24.2^d^	23.2	31.5^e^	68.5
20 t ha^−1^ TB	30.6^a^	54.7	47.5^b^	150.2
20 t ha^−1^ PM	26.8^b^	36	39.7^c^	110.4
TB + PM 10 t ha^−1^ each	31.6^a^	59.6	51.8^a^	172.2
10 t ha^−1^ TB	24.9^cd^	26,6	34.7^d^	84.8
10 t ha^−1^ PM	23.9^d^	21.7	33.1^de^	76.6
TB + PM 5 t ha^−1^ each	26.3^bc^	33.5	37.7^c^	100.2

Values followed by similar letters under the same column are not significantly different at p = 0.05 according to Duncan's multiple range test. NA = no amendment (control), TB = *Tithonia diversifolia* fresh biomass, PM = poultry manure, MF = Mineral fertilizer (450 kg ha^−1^ 13-13-23 NPK + 100 kg ha^−1^ Urea), ADB = aboveground dry biomass, FTY = fresh tubers yield, IR = improvement rate.

Correlation analysis in Table 8 revealed a negative and strong correlation between bulk density and growth and yield parameters, and positive and strong correlation between total porosity (TP), water holding capacity and growth and yield parameters. This means that growth and yield of cassava are extremely influenced by these soil physical properties.

**Table 8:** Correlation coefficient between growth and yield parameters of cassava and soil properties.

	FTY	ADB	SH	SD	CD
BD	-0.718∗∗	-0.660∗∗	-0.827∗∗	-0.679∗∗	-0.626∗∗
TP	0.716∗∗	0.658∗∗	0.824∗∗	0.674∗∗	0.627∗∗
WHC	0.741∗∗	0.689∗∗	0.841∗∗	0.635∗∗	0.673∗∗

BD = bulk density, TP = total porosity, WHC = water holding capacity, ADB = aboveground dry biomass, FTY = fresh tubers yield, SH = stem height, SD = stem diameter, CD = canopy diameter.

Note: ∗ Significant difference at p = 0.05, ∗∗ Significant difference at p = 0.01.

## 4 Discussion

Results have shown that the application of *Tithonia diversifolia* fresh biomass (TB) and poultry manure (PM) reduced soil bulk density and increased water holding capacity and porosity compared to the mineral fertilizer (MF) and the control treatments. The improvement of the bulk density, total porosity and water holding capacity in the plots treated with both organic fertilizers (TB and PM) could be related to the increase of organic matter, which improved soil structure and aeration, reduced soil bulk density, enhanced water infiltration and water content (Adekiya et al., 2017; Agbede et al., 2017; Hafifah et al., 2016). The general tendency of the decrease in soil bulk density observed by increasing the quatities of organic fertilizers applied during this study corroborates earlier findings by Kolawole et al. (2014) and Pravin et al. (2013), who noticed that the weight of the soil is strongly dependent on its level of soil organic matter content, and high application of organic manure reduce soil bulk density. The soil total porosity was increased with the application of TB and PM during the study. Indeed, the increase in soil total porosity with the application of organic fertilizers might be as a result of the improvement of soil particle aggregation brought by the increase in soil organic matter content (Agbede, 2021; Widowati et al., 2020). The soil water holding capacity was also enhanced with *Tithonia diversifolia* fresh biomass (TB) and poultry manure (PM) applied solely or combined. The results obtained agreed with the findings made by Sharma et al. (2021), who found an enhancement in soil physical properties such as water retention and aggregate stability under subtropical conditions due to the application of organic fertilizers. Hafifah et al. (2016) and Ewulo et al. (2008) earlier reported an improvement in water holding capacity of soil due to addition of organic fertilizers. Application of mineral fertilizer did not show any evidence of the improvement of soil physical parameters because of lack of organic matter, as reported earlier by Kolawole et al. (2014) and Hafifah et al. (2016).

The improvement of the growth and yield of cassava with *Tithonia diversifolia* fresh biomass (TB) and poultry manure (PM) applied soley or combined was a result of improved soil physical parameters such as bulk density, total porosity and water holding capacity. The poor performance of cassava recorded from the unamended plots (control) was probably due their soil physical conditions and their low content of soil organic matter. Plots treated with TB and PM solely or combined performed more than those treated with mineral fertilizer (MF) because of the enhanced soil physical conditions brought about by the application of organic fertilizers (Agbede et al., 2017; Agbede, 2021). In addition, organic manures are rich in fertilizing elements and can supply both macro- and micronutrients to the plant compared to the NPK fertilizer treatments (Adekiya and Agbede, 2017; Biratu et al., 2018a, Biratu et al., 2018b). Moreso, it has been reported that organic amendments application to acid soils reduce soil acidity and increase P availability contrary to mineral fertilizer (Fekadu et al., 2018; Haynes and Mokolobate, 2001). Consequently, soils treated with organic fertilizers respond better to crop production due to the enhancement of their quality.

Regardless of the cropping season, increasing the rate of organic fertilizers increased growth and yield of cassava such as stem height (SH), stem diameter (SD), canopy diameter, above ground dry biomass (ADB) and fresh tuber yield (FTY). In addition, the best performance in cassava growth and yield parameters were recorded on plots treated with TB and PM in the second cropping season (2017/2018). This was probably due to the accumulation effects after repeated addition of organic fertilizers. These findings were confirmed by those of Biratu et al., 2018a, Biratu et al., 2018b and Kolawole et al. (2014) who noticed increase on growth and yield parameters of cassava on further increase of different organic fertilizers. The best performance of cassava was recorded with the mixture of TB and PM applied at 10 t ha^−1^ each. This might be related to better soil physical conditions brought by this mixture of organic fertilizers. These results are consistent with observations by Agbede et al. (2017) who noted a significant improvement of yam yield due to the application of a mixture of green manure and poultry manure. Likewise, Al-Gaadi et al. (2019) in the central region of Saudi Arabia, has reported that crop yields performed better with the application of organic fertilizers such as poultry manure.

The study also revealed that cassava growth and yield parameters depended on soil physical and chemical properties. A positive and strong correlation was observed between growth and yield parameters of cassava and between total porosity and water holding capacity, Negative and strong correlation was noted between growth and yield parameters and bulk density, this indicated that the yield of cassava in the study site was strongly depend on soil physical parameters. In this study, our findings were similar to the earlier findings by Kolawole et al. (2014) and Agbede (2018) who noted that the performance of crops such as cassava was strongly affected by soil physical parameters, which might influence root penetration and nutrient uptake.

## 5 Conclusion

The study showed that application of organic fertilizers improved soil physical properties by lowering soil bulk density, and increasing total porosity and water holding capacity. Application of Tithonia and poultry manure either solely or mixed improved growth and yield parameters of cassava. Organic fertilizers performed better than mineral fertilizer. A general trend of increase in growth and yield parameters of cassava by increasing the proportions of organic fertilizers applied was observed. Combined application of *Tithonia diversifolia* fresh biomass and poultry manure at 10 t ha^−1^ each, was the optimum rate of producing cassava, which would support the demand placed on the soil to produce higher yield without deleterious effect on the crop and the environment. Organic manures can be used to improve cassava production and sustain soil productivity in the humid forest zone of Southern Cameroon.

## Declarations

### Author contribution statement

Eloi Gervais Bilong: Conceived and designed the experiments; Performed the experiments; Analyzed and interpreted the data; Wrote the paper.

Monique Abossolo-Angue and Paul Bilong: Conceived and designed the experiments; Wrote the paper.

Bienvenu Désiré Anaba: Performed the experiments; Contributed reagents, materials, analysis tools or data.

Francis Ngome Ajebesone; Birang À Madong and Lucien Bidzanga Nomo: Conceived and designed the experiments.

### Funding statement

This research did not receive any specific grant from funding agencies in the public, commercial, or not-for-profit sectors.

### Data availability statement

Data included in article/supp. material/referenced in article.

### Declaration of interests statement

The authors declare no conflict of interest.

### Additional information

No additional information is available for this paper.
